# Ultrasound promotes germination of aging *Pinus tabuliformis* seeds is associated with altered lipid metabolism

**DOI:** 10.1016/j.ultsonch.2023.106310

**Published:** 2023-01-23

**Authors:** Huahai Zhang, Weiyi Mo, Shaoming Liao, Zhongtao Jia, Wenjie Zhang, Shuoxin Zhang, Zhaojun Liu

**Affiliations:** a College of Forestry, Northwest A&F University, Yangling, Shaanxi 712100, China; b State-owned HouZhenZi Ecological Experimental Forest Farm of ZhouZhi County, Shaanxi 710000, China; c Key Laboratory of Plant-Soil Interactions, MOE, College of Resources and Environmental Sciences, National Academy of Agriculture Green Development, China Agricultural University, No. 2 Yuanmingyuan West Road, Haidian District, Beijing 100193, China; d Pu’er Institute of Pu-erh Tea, Yunnan 665000, China; e Qinling National Forest Ecosystem Research Station, Yangling, Shaanxi 712100, China; f Microelement Research Center, College of Resources & Environment, Huazhong Agricultural University, Wuhan 430070, China

**Keywords:** Ultrasound, Seed germination, *Pinus tabuliformis*, Lipidome, Transcriptome, Sphingolipid

## Abstract

Seed germination is an essential biological process for establishing new organisms of higher plants, it is especially significant for those aged seeds stored in gene banks for years. In this study, we investigated ultrasound treatment induced germination for aged *Pinus tabuliformis* seeds, which has been used in large scale aircraft sowing based afforestation in North China over 30 years’ ago without knowing possible mechanisms. We showed certain strength of ultrasound could increase the germination rate of aged seeds for about 3 times compare with control. Interestingly, although our transcriptome and lipidome analysis showed the differences between control and ultrasound treatments can be observed 1 day after germination by partial least squares discriminant analysis (PLSDA) analysis, majority (75 % or 69 %) of the significantly altered genes or lipids were commonly shared between them. Further analysis for the commonly altered lipids between both treatments showed ultrasound provoked the variations of lipids during germination process. Our investigation also revealed large amount of ultrasound-related genes and lipids that might be involved in germination promotion process. We hypothesis ultrasound provokes seed lipidome which further increases seed germination of *Pinus tabuliformis*. Our study provides new insides into the role of ultrasound induced lipidome change in seed germination. Moreover, we provide a new method to improve germination of aged seeds which might benefit preservation of seeds in gene banks.

## Highlights

• Certain strength of ultrasound enhanced the germination of 7 years’ naturally aged *Pinus tabuliformis* seeds for about 3 times;
• The germination process of *Pinus tabuliformis* seeds is associated with altered lipid metabolism according to transcriptome and lipidome analysis;
• Ultrasound provokes lipidome reprogramming during seed germination;

## 1 Introduction

*Pinus tabuliformis*, also known as Chinese red pine, is an native and dominant species of coniferous forest in North China [1]. It is an important tree species for afforestation with the purpose of soil conservation and timber supply, which is distributed in 16 Chinese provinces and autonomous regions with a land cover of over 2 million km^2^[2]. For its reproduction, *Pinus tabuliformis* depends mainly on seed which is also the choice for afforestation in North China [3].

Seed germination is a biological process finely regulated by internal and external factors to ensure successful seedling establishment under diverse conditions. Physical factors such as water-resistant seed coat is generally prevent germination, which could overcome by manual scarification or acid treatment [4]. Some pioneer forest tree species such as *Juniperus procera* and *Ficus lundellii*, their seed germination requires suitable light conditions, namely photo-dormancy [5], [6]. While above mentioned seed germination can be enhanced by certain physical or physiological regimes, natural or artificial aging caused low germination rate are generally irreversible. Although establishment of gene banks have been proved to be an efficient way of seed reservation, however, during the storage period, natural seed aging leads to reduced viability and the main cause are lipid peroxidation, enzyme inactivation, protein degradation and disruption of cellular membranes [7], [8]. In case of seed germination of Aleppo pine, lipase activity in lipid bodies were gradually increased, and treatments such as optimal concentration of NO3^–^/NH4^+^ which enhanced germination also coincided with enhanced lipase activity [9], suggesting improved germination might related with altered lipid metabolism. By contrast, knockout two carriers of small lipophilic molecules, temperature-induced lipocalin (TIL) and the chloroplastic lipocalin(CHL) which reduced mobilization of lipids, showed severely reduced germination after 4 days’ artificial aging, demonstrated the importance of lipid metabolism during seed germination [10]. Thus, lipid metabolism is important for seed germination.

Ultrasonication has been used in plant transformation to increase transformation efficiency over 30 years ago [11], [12]. The mechanically disrupts of the cell membranes by ultrasound wave is thought to permit a more efficient penetration of *Agrobacterium* into the explant tissues [13]. Actually, certain strength of ultrasound treatment also stimulated shoot regeneration [14] or callus growth [15], indicating it may stimulate physiological or even molecular processes rather than mechanically disruption of membranes. Using ultrasound treated *Pinus tabuliformis* seeds has been adopted in large scale aircraft sowing based afforestation in North China over 30 years’ ago, especially in short rainy season which requires fast germination in harsh conditions [16]. Studies showed ultrasound treatment promoted seed germination in diverse plant species including tall fescue [17], peanut [18] and rice [19], in which several germination-related enzymes including α-amylase increased their activities after ultrasoundation. Interestingly, another similar treatment, laser, can increase germination of Chinese pine seeds especially under drought condition, which is correlated with reduced malondialdehyde, a common product of lipid peroxidation [20]. These studies implied ultrasound could improve seed germination. Nevertheless, mechanisms of ultrasoundation in promoting seed germination is still elusive.

In this study, we adopted ultrasound to evaluate its effect on germination of aging *Pinus tabuliformis* seeds. Surprisingly, we found ultrasound promoted seed germination is associated with altered lipid metabolism according to our transcriptome and lipidome analysis. Our investigation found several lipid metabolism related genes as well as ultrasound-altered lipids belong to sub classes of sphingomyelin, digalactosyl monoacylglycerol and monoglyceride. Future studies for these genes or lipids could improve our understanding of ultrasound improved seed germination. To our best knowledge, this is the first study to report effect of ultrasound on seed germination with both transcriptome and lipidome approaches.

## 2 Materials and methods

### 2.1 Germination assay

The *Pinus tabulaefolia* seeds were collected in 2014 from Qinling National Forest Ecosystem Research Station, Shanxi Province, China, 33°18′–33°28′N, 109°20′-109°29′E, 1450–2500 m above the sea level. Seeds were stored at room temperature for 7 years. One hundred seeds of similar size without obvious damage were washed by Milli-Q water and randomly distributed to thin cotton bags. For control, cotton bags with seeds were placed in ultrasound cleaner with Milli-Q water for 40 min without ultrasound treatment, while ultrasound treatment followed the same procedure and in addition with ultrasound treatment (KQ-500DE, Kunshan Ultrasonic Instrument 40 KHz, 100 % amplitude) for 40 min. All treatments were placed in a growth chamber maintained at temperature 25 °C. After 40 min, seeds of both treatments were placed on wet filter paper and arranged on a plastic tray. 100 seeds/ tray. All trays were placed in a climate chamber with constant temperature of 25 °C, 80 % humidity without light for 18 days. Seed germination was examined every day, and germination is defined as opening of the seed coat with visible emergence of root. Root length was measured by a ruler at day 18.

### 2.2 Transcriptome analysis

Samples for transcriptome analysis were collected directly after treatments (day 0) or after 1 day’s germination (day 1) in the climate chamber. The harsh seed coats were quickly removed then freeze in liquid nitrogen. Homogenized samples were subjected for RNA isolation and samples were quantified by a NanoDrop spectrophotometer (Thermo Scientific) and Agilent 2100 bioanalyzer. Samples with RIN (RNA integrity number) value ≥ 7 were sending for sequencing. Brief steps for generating sequencing libraries: mRNA was purified from total RNA by poly-T oligo-attached magnetic beads and randomly interrupted using divalent cations. Then fragmented mRNA was reversed and purified to cDNA. After adenylation of the 3′ ends of the cDNA fragments, Illumina PE adapter oligonucleotides were ligated to prepare. Finally, used AMPure XP (Beckman Coulter,Beverly, CA, USA) beads to selected qualified cDNA to PCR amplification. Products were purified (AMPure XP system) and quantified using the Agilent high sensitivity DNA assay on a Bioanalyzer 2100 system (Agilent). The sequencing library was then sequenced on NovaSeq 6000 platform (Illumina) by BGI Genomics.

### 2.3 Quantitative Real-time PCR

Total RNA was extracted using Trizol reagent (TRIpure Reagent, Aidlab, China). Then, the concentration and absorbance of RNA were measured by NanoDrop 2000c (Thermo Scientific, Massachusetts, USA). For each sample, cDNA was synthesized based on 1000 ng of total RNA by the Reverse Transcription kit (TRUEscript RT Kit, Aidlab, China). For quantitative real-time PCR, 2x Universal SYBR Green Fast qPCR Mix (ABclonal, China) was applied on CFX96TM Real-time PCR Detection System (Bio-Rad, Hercules, CA, USA). Five biological replications were used for each treatment and three technical replications for each sample. *Pinus tabulaefolia* elongation factor 1 (EF1) was used as the reference gene, and all primers were listed in Supplementary Table 20. Gene expression were calculated according to the 2^-ΔΔCt^ method.

### 2.4 Lipidome analysis

800 µl extraction buffer (dichloromethane: methanol = 3:1, v/v, −20 °C) and 10 µl SPLASH Internal standards (330707, SPLASHTM Lipidomix Mass Spec Standard, Avanti Polar Lipids, USA) were mixed with 25 mg samples for further homogeneous grinding (50 Hz, 5 min), followed by 10 min ultrasound treatment in 4 °C water bath and 1 h stabilization in −20 °C. Samples were centrifuged at 25000 rpm, 4 °C for 15 min, and 600 µl supernatant of each sample were transferred to a new EP tube for freeze dry. Dry samples were dissolved by 200 µl buffer (sopropanol: acetonitrile: water = 2: 1: 1, v/v/v), dissolving was assisted by 1 min vortex and 10 min ultrasound treatment in 4 °C water bath. The solution was again centrifuged at 25000 rpm, 4 °C for 15 min, and the supernatant was ready for UPLC-MS analysis.

CSH C18 columns (1.7 μm 2.1*100 mm, Waters, USA) were used in the UPLC-MS quantification. The mobile phase consisted of solvent A (60 % acetonitrile aqueous solution + 0.1 % formic acid + 10 mM ammonium formate) and solvent B (10 % acetonitrile aqueous solution + 90 % Isopropanol + 0.1 % formic acid + 10 mM ammonium formate) under positive ion mode. The solvent A (60 % acetonitrile aqueous solution + 10 mM ammonium formate) and solvent B (10 % acetonitrile aqueous solution + 90 % Isopropanol + 10 mM ammonium formate) under negative ion mode. Gradient elution conditions were set as follows: 0 ∼ 2 min, 40 % to 43 % solvent B; 2 ∼ 2.1 min, 43 % to 50 % solvent B; 2.1 ∼ 7 min, 50 % to 54 % solvent B; 7 ∼ 7.1 min, 54 % to 70 % solvent B; 7.1 ∼ 13 min, 70 % to 99 % solvent B; 13 ∼ 13.1 min, 99 % to 40 % solvent B; 13.1 ∼ 15 min, 40 % solvent B. The flow rate was 0.35 mL/min. The column oven was maintained at 55 °C. The injection volume of each sample was 5 μl.

## 3 Statistics

All statistical data in this study were analysis with Student's *t*-test (* *P* < 0.05, ** *P* < 0.01, *** *P* < 0.001) by using the SigmaPlot software 14.0 (Systat Software Inc., Germany).

## 4 Results

### 4.1 Ultrasound treatment promotes germination of aging *Pinus tabuliformis* seeds

**Fig. 1:**
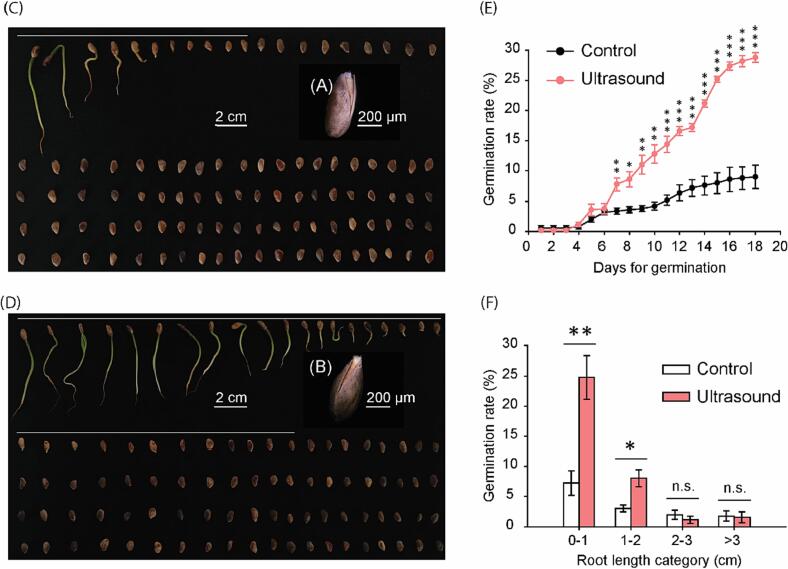
**Ultrasound treatment promoted germination of aging *Pinus tabuliformis* seeds.** Seed images at early germination time of control (A) or ultrasound (B) treatments. Representative germination overview of control (C) or ultrasound (D) at day 18. (E) The germination rates at each day of both treatments overtime. (F) The distribution of the germination rates of different root length categories in both treatments. 7 years’ old natural aging seeds of *Pinus tabuliformis* were used in this study. Control treatment: seeds were soaked in Milli-Q Water in ultrasound cleaner without ultrasound treatment for 40 min. Ultrasound treatment: seeds were soaked in Milli-Q Water in ultrasound cleaner with ultrasound treatment (40 KHz, 100 % amplitude) for 40 min. White lines in (A) and (B) above seeds outline the germinated individuals in the treatments. The 2 cm bars correspondent to the photographs of the 100 seeds of each treatment, and the 200 µm bars represent the standards for the germinated single seeds in each treatment. Each treatment has 5 replications and each replication contains 100 seeds. Values represent mean ± SE. ***P < 0.001,**P < 0.01, *P < 0.05, n.s.: not significant.

To illustrate effects of ultrasound on germination of aging seeds, 7 years’ old *Pinus tabuliformis* seeds collected in 2014 from Qinling National Field Research Station of Forest Ecosystem (Shanxi Province, China, 33°18′–33°28′N, 109°20′-109°29′E, 1450–2500 m above sea level) and preserved at room temperature with natural aging process were used in this study. Seeds soaked in Milli-Q water in the ultrasound washing machine under turned off mode for 40 min referred as control treatment, while in the same condition with turned on mode and 100 % amplitude was considered as ultrasound treatment. We define germination of the *Pinus tabuliformis* seeds as opening of the seed coat with visible emergence of root (Fig. 1A, B). After 18 days’ germination, we saw control treatment had about 10 % germination, while ultrasound treatment promoted the germination to ∼ 30 % (Fig. 1C, D). Moreover, seedlings of ultrasound treatment has longer shoots compared with control, suggesting ultrasound could also promote shoot growth directly or indirectly.

To examine the seed germination with higher resolution, we counted germination rate each day until day 18. Our data showed there was no statistical difference in germination rate until day 7, after which ultrasound significantly increased germination. This difference was enlarged until day 18 when germination rate was stable (Fig. 1E). At day 18, germination of ultrasound treatment was about 3 times than that of control. Detailed examination of germinated seeds showed ultrasound promoted germination rate of root length categories of 0–1 and 1–2 cm, but not the other categories (Fig. 1F).

### 4.2 Transcriptome profiling and differentially expressed genes between control and ultrasound treatments

**Fig. 2:**
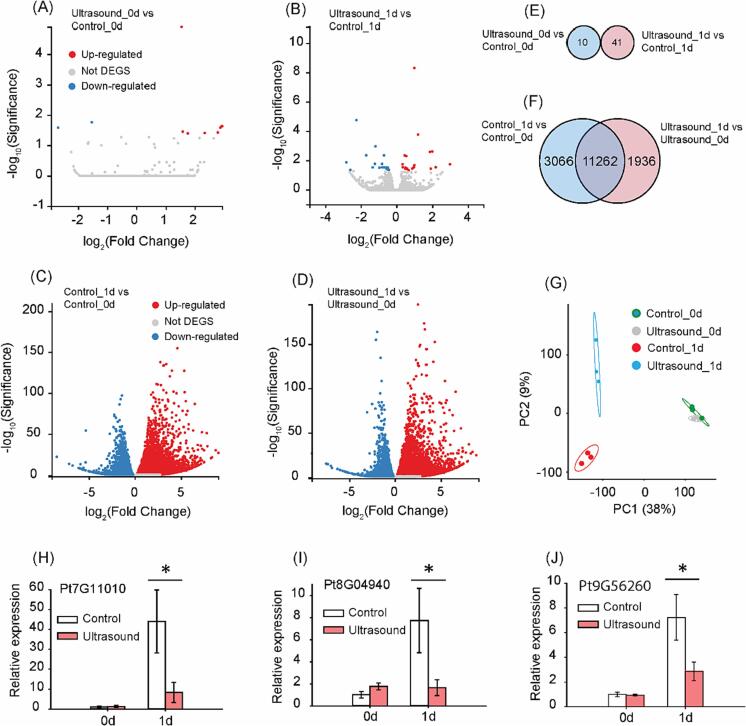
**Transcriptome analysis for control and ultrasound treated *Pinus tabuliformis* seeds.** Differentially expressed genes (DEGs) in the comparisons of ultrasound_0d vs control_0d (A), ultrasound_1d vs control_1d (B), control_1d vs control_0d (C) and ultrasound_1d vs ultrasound_0d (D). Venn diagrams of ultrasound_0d vs control_0d/ ultrasound_1d vs control_1d (E), and control_1d vs control_0d/ ultrasound_1d vs ultrasound_0d (F). (G) Partial least squares discriminant analysis (PLSDA) analysis for the transcriptome of control and ultrasound treated seeds at day 0 and day 1. qPCR analysis for Pt7G11010 (H), Pt8G04940 (I) and Pt9G56260 (J) in control and ultrasound treatments. Control treatment: seeds were soaked in Milli-Q Water in ultrasound cleaner without ultrasound treatment for 40 min. Ultrasound treatment: seeds were soaked in Milli-Q Water in ultrasound cleaner with ultrasound treatment (40 KHz, 100 % amplitude) for 40 min. Definition of DEGs in the transcriptome: False Discovery Rate (FDR) < 0.05. For transcriptome and qPCR, n = 3. *P < 0.05.

To understand early transcriptome changes after ultrasound treatment, we collected seed samples at day 0 and day 1 (each treatment with 3 biological replications), and subjected them for RNA-Seq analysis. We use newly available genome sequencing data of *Pinus tabuliformis* as the reference for our transcriptome results [21]. Throughout our transcriptome analysis, we use adjusted p (also named as q value) < 0.05 as the cutoff to define differentially expressed genes (DEGs). As expected, there were only handful genes significantly altered between ultrasound and control at day 0 or day 1 (Fig. 2A, B, Supplementary Tables 1,2), because significant germination difference was only observed after 7 days’ germination (Fig. 1E). Thus, day 0 and day 1 were still at very early developmental stages with limited transcriptome changes. We then focused on long terms effect of ultrasound on germination, to evaluate the effect of ultrasound after 1 day’s germination. In this case, there were over 10,000 genes identified as DEGs in the comparison of control_1d vs control_0d or ultrasound_1d vs ultrasound_0d, respectively (Fig. 2C, D, Supplementary Tables 3,4).

To dissect short-term and long-term ultrasound altered genes, we compared DEGs between different comparisons, including: 1) ultrasound_0d vs control_0d and ultrasound_1d vs control_1d; 2) control_1d vs control_0d and ultrasound_1d vs ultrasound_0d. Surprisingly, only 10 or 41 DEGs can be identified in the first comparison (Fig. 2E, Supplementary Tables 1,2), suggesting ultrasound had mild effect for gene expression. It is worth noting that there was no DEG shared between ultrasound_0d vs control_0d and ultrasound_1d vs control_1d, implied ultrasound effects at day 0 and day 1 were different. In case of long-term ultrasound effect, we identified 14,328 or 13,198 DEGs in the comparison of control_1d vs control_0d or ultrasound_1d vs ultrasound_0d, respectively (Fig. 2F). Interestingly, majority of the DEGs (69 %, 11,262 out of 16,264 DEGs) were commonly shared between control_1d vs control_0d and ultrasound_1d vs ultrasound_0d, and only 3066 or 1936 DEGs were uniquely identified in each comparison (Fig. 2F). This result implied ultrasound seems only had mild effect at early germination stage.

To have an overview of the transcriptome data, we conducted partial least squares discriminant analysis (PLSDA) for our dataset. Our analysis was not able to separate control and ultrasound samples of day 0 (Fig. 2G), indicating ultrasound only induced mild transcriptome changes immediately after the treatment. However, after one day’s germination, samples of the control and ultrasound treatments were clearly separated according to the 95 % confidence ellipses, suggesting a stronger transcriptome alteration after ultrasound treatment over one day’s germination. Taken together, the transcriptome result suggested although ultrasound had mild effect in short-term condition, long-term effect of ultrasound at transcriptome level was profound, even there was no clear germination difference at day 1.

### 4.3 qPCR verification for expressions of ultrasound altered genes

We further validated few of the significantly altered genes from the transcriptome analysis. Since the genome of *Pinus tabuliformis* was just sequenced and large amount of the genes were poorly annotated [20], we choose 3 significantly altered genes (from the comparison between ultrasound_1d vs control_1d) with clear annotation from NCBI (https://www.ncbi.nlm.nih.gov/) for qPCR verification. We saw Pt7G11010 (a dehydrin gene), Pt8G04940 (a pyruvate dehydrogenase gene) and Pt9G56260 (a 60S ribosomal gene) maintained their expressions in case of ultrasound_0d vs control_0d (Fig. 2H, I, J). At day 1, all 3 genes were up-regulated compared with day 0 except Pt8G04940, and the strongest upregulation were detected at control treatment, suggesting ultrasound suppressed the expression of these 3 genes in a long-term effect.

### 4.4 KEGG and GO enrichment analysis showed lipid metabolism is associated with germination and ultrasound treatments

To better understanding possible biological processes altered by ultrasound, we first did clustering analysis and showed these DEGs can be categorized into 12 clusters with diverse expression patterns (Supplementary Figure 1). We then subjected genes of each cluster for Kyoto Encyclopedia of Genes and Genomes (KEGG) analysis and use criteria of p < 0.05 for defining significantly enriched terms. The KEGG approach revealed enrichment of diverse biological processes from these 12 clusters. Interestingly, several lipid metabolism related terms frequently appeared in 9 of the 12 clusters except cluster 7, 9 and 11 (Supplementary Tables 5-16), including fatty acid metabolism, glycerolipid metabolism and sphingolipid metabolism, suggesting a profound involvement of lipid metabolism during germination and ultrasound treatment. These results suggested ultrasound might altered lipid metabolism during seed germination.

**Fig. 3:**
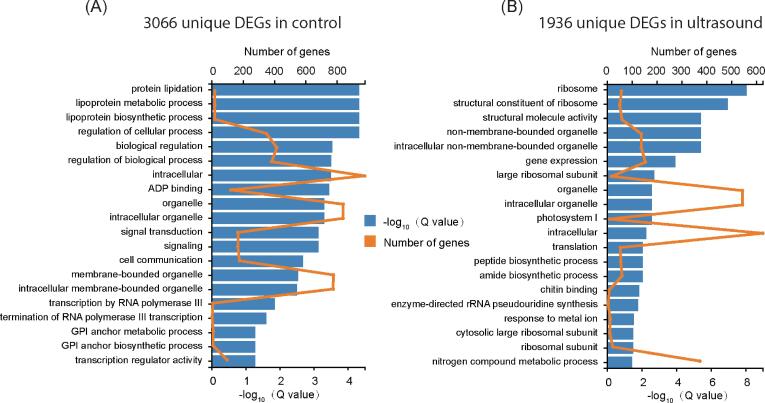
**Gene ontology (GO) analysis for the uniquely differential expressed genes (DEGs) in control or ultrasound treatments**. (A) Top20 GO terms of the 3066 unique DEGs in control treatment over 1 day. (B) Top20 GO terms of the 1936 unique DEGs in ultrasound treatment over 1 day. The × axis stands for –log_10_ transformed q value of GO term (blue column) or number of genes (orange line) in the GO term. The y axis stands for the names of the top20 GO terms in each enrichment analysis. Definition of DEGs in the transcriptome: False Discovery Rate (FDR) < 0.05. Threshold for the enrichment of GO terms: p value < 0.05. (For interpretation of the references to colour in this figure legend, the reader is referred to the web version of this article.)

We are especially interested to know ultrasound or control specific biological processes. According to our gene ontology (GO) analysis and cutoff of p < 0.05, 325 or 259 GO terms were enriched by the 3066 or 1936 unique DEGs, respectively (Supplementary Tables 17,18). Displaying the top 20 GOs of each groups showed several cell metabolic activity related processes were enriched over 1 day’s treatments, including terms like regulation of cellular process, transcription by RNA polymerase III, ribosome and translation, implying reprogramming of cell metabolism after 1 day’s germination (Fig. 3A, B). Interestingly, GO terms like protein lipidation, lipoprotein metabolic process and lipoprotein biosynthetic process were enriched in the group of 3066 DEGs. In comparison, several transcription related terms like ribosome, gene expression and translation were enriched in the group of 1936 DEGs. GO enrichment for both groups suggested active cellular metabolism and transcription were happening during seed germination. Moreover, several lipid metabolism related terms together with previous publications encouraged us to investigate lipidome changes during seed germination, especially after ultrasound treatment.

### 4.5 Lipidome analysis for germination of aging *Pinus tabuliformis* seeds

**Fig. 4:**
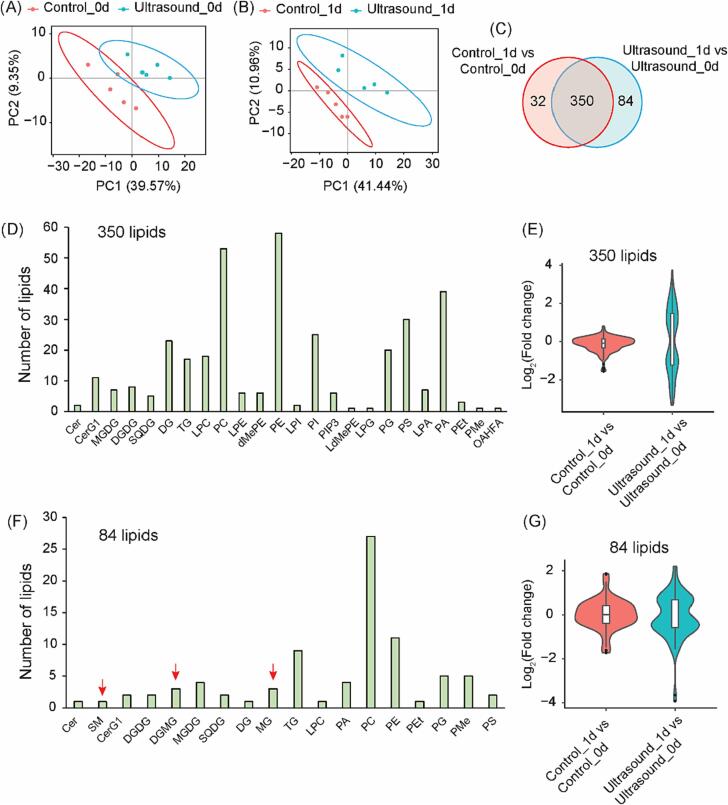
**Lipidome analysis for control and ultrasound treated *Pinus tabuliformis* seeds.** Partial least squares discriminant analysis (PLSDA) analysis for the lipidome of control and ultrasound treated seeds at day 0 (A) and day 1 (B). (C) Venn diagram of unique or common differentially altered lipids (DALs) between control and ultrasound treatments over 1 day. (D) Classification of 350 common DALs between control and ultrasound treatment over 1 day. (E) Overview of the variations of the 350 common DALs between control and ultrasound treatments over 1 day. (F) Classification of 84 unique DALs of ultrasound treatment over 1 day. (G) Overview of the variations of the 84 unique DALs of ultrasound treatment over 1 day. Cer: Ceramides; CerG1: Monogylcosyl-ceramide; MGDG: Monogalactosyl-diacylglycerol; DGDG: Digalactosyl-diacylglycerol; DGMG: Digalactosyl-monoacylglycerol; SQDG: Sulfoquinovosyl-diacylglycerol; DG: Diglyceride; TG: Triglyceride; LPC: Lyso-phosphatidylcholine; PC: Phosphatidylcholine; LPE: Lyso-phosphatidylethanolamine; dMePE: Dimethylphosphatidylethanolamine; PE: Phosphatidylethanolamine; LPI: Lyso-phosphatidylinositol; PI: Phosphatidylinositol; PIP3: Phosphatidylinositol (3,4,5)-trisphosphate; LdMePE: Lyso-dimethyphosphatidylethanolamine; LPG: Lyso-phosphatidylglycerol; PG: Phosphatidylglycerol; PS: Phosphatidylserine; LPA: Lyso-phosphatidic acid; PA: Phosphatidic acid; PEt: Phosphatidylethanol; PMe: Phosphatidylmethanol; OAHFA: (O-acyl)-1-hydroxy fatty acid; SM: Sphingomyelin; MG: Monoglyceride. Definition DALs: False Discovery Rate (FDR) < 0.05. The red arrows in (F) indicate 3 lipid sub classes altered by ultrasound. (For interpretation of the references to colour in this figure legend, the reader is referred to the web version of this article.)

Due to the indications of altered lipid metabolism processes from the transcriptome analysis, we subjected the samples for lipidome analysis and each treatment with 5 biological replications. In total, there were 540 lipids identified in the seed samples (Supplementary Table 19). Similar to the transcriptome results, the PLSDA analysis for lipidome could not separate samples of control and ultrasound treatments at day 0, but successfully differentiated these two treatments at day 1 according to 95 % confidence ellipses (Fig. 4A, B), suggesting a significant alteration by ultrasound treatment after one day. Surprisingly, when comparing ultrasound_0d vs control_0d or ultrasound_1d vs control_1d, none of these 540 lipids showed significant change (Supplementary Table 19), although our PLSDA analysis could at least separate samples between ultrasound_1d and control_1d (Fig. 4B). Nevertheless, this result resembles the transcriptome analysis when comparing ultrasound_0d vs control_0d or ultrasound_1d vs control_1d, which resulted with only 10 or 41 genes as DEGs. Taken together, these data implied ultrasound has mild effect for short-term seed germination.

We thus focus on long-term effect of ultrasound by comparing control_1d vs control_0d and ultrasound_1d vs ultrasound_0d, and identified about 75 % (350 out of 466) differentially altered lipids (DALs) were commonly identified in both comparisons (Fig. 4C). This value is close to 69 %, the proportion of the DEGs commonly identified in both comparisons (Fig. 2F). Although majority of the DALs were commonly altered in both treatments, there were 32 or 84 lipids identified as unique DALs in both comparisons, respectively (Fig. 4C). We further categorized these 350 commonly altered DALs into 24 sub classes (Fig. 4D), which implied a wide alteration of lipidome during natural seed germination as well as ultrasound treatment. Ranking the number of lipids in each sub class, the top5 sub classes of these 350 DALs have been identified as PE (phosphatidylethanolamine), PC (phosphatidylcholine), PA (phosphatidic acid), PS (phosphatidylserine) and PI (phosphatidylinositol), each with 58/ 53/ 39/ 30/ 25 lipids within the classes, respectively (Fig. 4D). We transformed the abundance changes of the DALs by log_2_ method, and our result showed these 350 DALs showed much stronger variations due to ultrasound treatment (Fig. 4E). In addition, the 84 unique DALs altered by ultrasound can be distributed into 18 sub classes, and the top5 sub classes are PC, PE, TG (triglyceride), PG (phosphatidylglycerol) and PMe (phosphatidyl methanol), each with 27/ 11/ 9/ 5/ 5 lipids within the classes, respectively (Fig. 4F). Interestingly, although these 84 lipids were not significantly altered during 1 day’s control treatment, they also showed much higher variations like the case of commonly altered 350 lipids (Fig. 4G).

It is worth noting that due to ultrasound treatment, several lipids belong to 3 lipid sub classes including SM (sphingomyelin), DGMG (digalactosyl monoacylglycerol) and MG (monoglyceride) were significantly altered (Fig. 4F). Lipids belong to these 3 sub classes were not identified in the control comparison, suggesting they are specific to ultrasound treatment. The IDs of these lipids are: 5.46_703.57486355 (SM sub class), 1.04_723.38087385/ 0.9_721.3652573/ 1.28_725.39656815 (DGMG sub class) and 2.67_455.40987715/ 2_374.3264291/ 2_357.29981155 (MG sub class). Further investigation of these lipids might extend our understanding of ultrasound improved seed germination. Taken together, the correlation of ultrasound treatment and increased alteration of lipid concentrations suggested ultrasound treatment might influence lipid metabolism to promote seed germination.

## 5 Discussion

Ultrasound has been used in food science to improve oil extraction from plant or animal organisms, which also changes lipid composition during the processing [22], [23]. It was reported that low-intensity ultrasound significantly affect capacitance of lipid bilayers [24]. In case of *Escherichia coli* (*E. coli*), strong ultrasound inhibited their growth and biological processes related to membrane lipid metabolism were significantly enriched after ultrasound treatment, suggesting ultrasound has a profound effect for lipidome [25]. During seed germination, seed lipidome undergo a certain metabolism processes including degradation and remobilization to sustain a proper germination, and blocking lipase activity or lipid mobilization by fungal infection impaired rice seed germination [26]. These studies implies ultrasound could affect seed germination via influencing seed lipidome. Indeed, our transcriptome analysis showed ultrasound could enrich several lipid metabolism related KEGG terms (Supplementary Tables 5-16). This observation was further supported by the seed lipidome analysis, and ultrasound altered more lipids than control treatment (Fig. 4C). Thus, our transcriptome and lipidome analysis demonstrated ultrasound could manipulate seed lipidome metabolism during germination.

To promote germination of aging seeds is important for gene banks, since long-term stored seeds will eventually lose their viability [27]. In fact, using ultrasound treatment to increase the germination of woody plants in aircraft sowing based afforestation has been used in North China over 30 years ago [16], but the mechanism remains unclear. In our transcriptome analysis, we documented ultrasound could alter lipid metabolism related biological processes and verified the expressions of some genes that could be annotated in NCBI. These are Pt7G11010/ Pt8G04940/ Pt9G56260 encode dehydrin protein/ pyruvate dehydrogenase protein/ 60S ribosomal protein, respectively (Fig. 2H, I, J). Dehydrins are late embryogenesis abundant (LEA) proteins that accumulate during seed maturation, and one of the functions of dehydrins is to be involved in stress response [28]. Interestingly, overexpress a citrus dehydrin protein in tobacco leads to increased cold tolerance and reduced peroxidation of liposomes [29], suggesting possible involvement of dehydrin proteins in lipid metabolism. Pyruvate dehydrogenase proteins are involved in tricarboxylic acid cycle by synthesizing Acetyl-coenzyme A, which is an important precursor during lipid accumulation in developing seeds [30]. Upregulation of pyruvate dehydrogenase E1a subunit results with elevation in seed storage oil contents [31]. Interestingly, the pyruvate dehydrogenase gene (Pt8G04940) was significantly altered in control but not by ultrasound, suggesting ultrasound changed seed lipidome might partially by suppressing the expression of pyruvate dehydrogenase. The down-regulated 60S ribosomal protein by ultrasound implied ultrasound might alter protein synthesize during seed germination via ribosome activity.

It is very interesting that 7 lipids belong to 3 lipid sub classes were significantly altered due to ultrasound treatment, which were not changed by control treatment (Fig. 4F). One of the seven lipids, 5.46_703.57486355, is a sphingomyelin belongs to the main class of sphingolipids. Knockout Arabidopsis orosomucoid-like (ORM) genes like ORM1 and ORM2 leads to unregulated sphingolipid biosynthesis which also associated with strongly reduced seed germination [32], suggesting the important roles of sphingolipid in germination process. The sphingolipid (ID: 5.46_703.57486355) was significantly altered by ultrasound but not by control treatment, suggesting ultrasound might alter this sphingolipid to promote germination. There were three DGMG lipids, 1.04_723.38087385/ 0.9_721.3652573/ 1.28_725.39656815, were significantly altered by ∼ 3.3/ 3.1/ 1.8-fold in ultrasound treatment but not in control (Supplementary Table 19). In case of cold stress, cold-tolerant wheat cultivar young accumulate higher concentration of DGMG lipids than cold-sensitive cultivar Wyalkatchem, which could be associated with the acylation response during cold stress and related to col-tolerant mechanism [33]. In addition, some lipids of DGMG have antioxidant capacity [34]. Thus, we suspect these ultrasound treatment increased DGMGs might be involved in antioxidant process during germination. The other three monoglycerides that only altered by ultrasound, 2.67_455.40987715/ 2_374.3264291/ 2_357.29981155, were ∼ 1.9/ 0.61/ 0.57-fold altered (Supplementary Table 19). To our best knowledge, there is no report about the biological roles of these monoglycerides during seed germination. Future study for these 7 ultrasound triggered lipids might explore mechanisms of ultrasound improved seed germination.

In our control treatment, we identified 382 DALs out of 540 lipids during *Pinus tabuliformis* seed germination without ultrasound treatment, suggesting a profound lipid metabolism process was happening. Surprisingly, ultrasound only significantly altered 84 lipids during germination compared with control, and 350 DALs were commonly altered between control and ultrasound treatments (Fig. 4C). Investigating of these two lipid groups revealed a trend that ultrasound treatment tended to reinforce the variations to a larger extend (Fig. 4E, G), suggesting a stronger lipid alteration process might function as a dominant mechanism in seed germination. In fact, plants do reprogram organ lipidome in order to adapt to varies stress scenarios including salt [35] and heat stresses [36]. In case of seed germination, knockout Arabidopsis caleosin-1 (*AtACLO-1*), which involves in the degradation of oil-bodies during seed germination, significantly retarded seedling growth at early stage [37]. Similar results were obtained by suppressing expression of rice lipid transfer protein 36 (*OsLTPL36*), which leads to reduced fat acid content in the seeds and lower germination rate [38]. It was suspected that ultrasound induced mechanical damage might allow the release of intracellular enzymes which cause damage on the chain structure of the lipids [39], which in our case might lead to enlarged alteration of certain lipid groups. In which way ultrasound manipulates lipid metabolism needs more studies. According to our findings, we propose ultrasound reinforced existing lipidome metabolism processes during seed germination, which finally increased germination of *Pinus tabuliformis* seeds. Our finding also demonstrates a strategy to increase germination of aging seeds, especially for those endangered species with limited seeds in gene banks.

## CRediT authorship contribution statement

**Huahai Zhang:** Conceptualization, Investigation. **Weiyi Mo:** Methodology, Investigation. **Shaoming Liao:** Resources. **Zhongtao Jia:** Data curation. **Wenjie Zhang:** Methodology. **Shuoxin Zhang:** Conceptualization, Supervision. **Zhaojun Liu:** Conceptualization, Supervision, Funding acquisition, Data curation, Writing – original draft, Writing – review & editing.

## Declaration of Competing Interest

The authors declare that they have no known competing financial interests or personal relationships that could have appeared to influence the work reported in this paper.
